# Severe aplastic anemia preceding acute monocytic leukemia in an adult with acquired trisomy 21: A case report

**DOI:** 10.3892/ol.2013.1724

**Published:** 2013-12-03

**Authors:** DONGMEI GUO, QINQIN LIU, BANBAN LI, QINGLIANG TENG

**Affiliations:** Department of Hematology, The Central Hospital of Taian, Taian, Shandong 271000, P.R. China

**Keywords:** severe aplastic anemia, leukemia, trisomy 21

## Abstract

The current case report presents a patient with acute monocytic leukemia (AML-M5) occurring 14 years following the successful treatment of severe aplastic anemia (SAA) with immunosuppressants and androgens. The patient was treated with induction chemotherapy, but did not achieve remission. The patient succumbed to central nervous system bleeding 2 weeks following the first cycle of chemotherapy. Chromosomal examination revealed 47,XX,+21[10]/46,XX[1]. To the best of our knowledge the present case is the first to be reported of SAA 14 years preceding AML-M5 with acquired trisomy 21.

## Introduction

Aplastic anemia (AA) is a clinical syndrome of peripheral blood pancytopenia and a hypocellular bone marrow. Immunosuppressive therapy is a key treatment strategy for AA. Genomic instability in AA does not appear to be a rare event. AA evolves into acute myeloid leukemia (AML) in 5–15% of all cases (1,2). Careful observation for leukemic transformation is indicated in patients with severe AA (SAA). Trisomy 21 is relatively common in Down syndrome, but rare in secondary AML. The current report presents the first case of SAA preceding acute monocytic leukemia (AML-M5) with acquired trisomy 21. Written informed consent was obtained from the patient’s family.

## Case report

A 20-year-old female was admitted to the Department of Hematology, The Central Hospital of Taian (Taian, China) in May 1996 with fever, dizziness and increasing tiredness. The patient exhibited no other positive medical or family history of any specific disease. In addition, the patient did not exhibit any features of Down syndrome. On physical examination, the patient was severely ill, pale and covered with mucocutaneous petechial bleeding. No lymphadenopathy or hepatosplenomegaly was observed. Viral serology was negative for hepatitis A, B and C, as well as human immunodeficiency virus. On full blood count, hemoglobin (Hb) count was 78 g/l, white blood cell count was 0.7×10^9^/l, platelet count was 16×10^9^/l, neutrophil count was 0.46×10^9^/l and reticulocyte count was 0.6%. Bone marrow aspirate was profoundly hypocellular and no megakaryocytes were observed. No mast cells, but a few foci of plasma cells and lymphocytes were identified. Residual myeloid and erythroid precursors were observed without any abnormal infiltrate. The patient was diagnosed with SAA.

The individual was treated with intravenous broad-spectrum antibiotics and multiple red cell and platelet transfusions with resolution of the fever, dizziness and increasing tiredness. The patient received, with high-dose methylprednisolone, cyclosporine A, androgens and recombinant human granulocyte colony-stimulating factor (G-CSF). Two years later, the blood counts returned to normal and the patient gradually stopped receiving drug therapy.

In 2001, the patient delivered a normal child. However, the results of the patients routine blood test were abnormal; the platelet count was <10×10^9^/l and Hb levels were also decreased. The patient received blood and platelet transfusions without other drug treatment.

**Figure 1:**
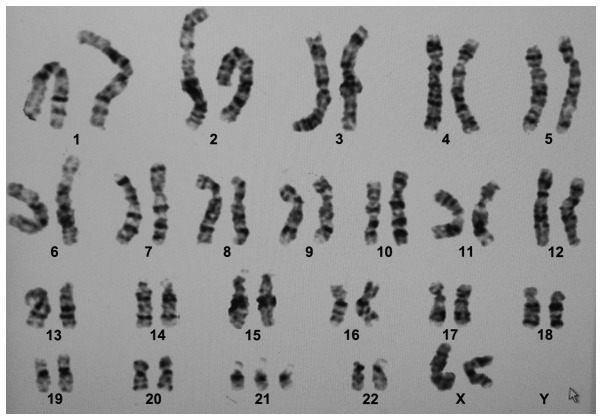
Chromosomal analysis.

In October 2010, the patient was hospitalized due to nasal bleeding and mild headache. On full blood count, white blood cell count was 19.92×10^9^/l, neutrophil count was 5.89×10^9^/l, monocyte count was 2.09×10^9^/l, Hb count was 105 g/l, platelet count was 20×10^9^/l and the reticulocyte count was 4.27%. Blast cells were observed on blood film and the bone marrow smears showed an increased number (31%) of monoblasts and promonocytes. Blast cells stained negatively for PAS and specific esterase stain and positive for non-specific esterase, which was inhibited by sodium fluoride. These observations were compatible with AML-M5b and chromosomal examination revealed 47,XX,+21[10]/46,XX[1] (Fig. 1). The patient was treated with induction chemotherapy, but did not achieve remission. The patient succumbed to central nervous system bleeding 2 weeks following chemotherapy.

## Discussion

Leukemia is a rare and late complication of AA. AML and myelodysplasia syndromes (MDS) occur in 15% of patients within 10 years following immunosuppressive treatment (3). There have been various reasons proposed as to why AA may precede AML. Previously, Barrios *et al*(4) presented a patient with SAA, with no previous chromosomal abnormalities, who developed trisomy 21 and monosomy 7 during treatment with intravenous cyclosporine. The abnormal karyotype disappeared when the drug was changed to the oral form. Furthermore, the published data on the safety of G-CSF for AA remain controversial. It has been reported that G-CSF therapy does not increase the risk of t-MDS/AML development (5–7). However, specific results have shown that G-CSF therapy is one of the risk factors for AA evolution to MDS/AML (8–10). Further studies are required to identify the risk factors in SAA for developing MDS/AML.

The evolution of AA to clonal hematologic diseases is well recognized. Cytogenetics are usually normal in the aplastic phase, but abnormalities may develop in the leukemic phase. The current report presents a new case of SAA preceding AML with trisomy 21 as the sole acquired karyotypic abnormality.

Trisomy 21 has been demonstrated to be a recurring cytogenetic abnormality in AML and MDS. Trisomy may contribute to leukemogenesis by a gene dosage effect. The majority of adult AML cases with trisomy 21 have been associated with AML-M2 or -M4 (11). By comparison, the most common hematological malignancy in patients with Down syndrome is AML-M7 (8,12). The prognostic significance of acquired trisomy 21 as the sole abnormality in adult AML remains unclear. A higher complete remission has previously been reported in AML with trisomy 21 (13), but a study showed that other accompanied cytogenetic changes determined the clinical outcome (14). In addition, a greater number of studies have shown that AML patients with acquired trisomy 21 as the sole abnormality exhibit a poor prognosis (14–16). Therefore, further studies with larger cohorts of patients are required to evaluate the prognostic significance of acquired trisomy 21.

The course and prognosis of secondary leukemia depends on not only cytogenetic features, but also clinical and molecular features at diagnosis. Previously, the successful therapy of such secondary leukemia has been rarely reported. Patients have not responded or have succumbed to infection or bleeding during induction. Irreversible aplasia is a hazard of intensive chemotherapy. Fatal bleeding events eventually occurred in the present patient, who was refractory to platelet transfusions.

Cytomorphological and cytogenetic abnormalities are rarely observed in AA, which may predict patients with SAA at risk for leukemia. Prior to the diagnosis of AML, SAA patients may develop signs of MDS. Therefore, long-term follow-up is essential to assess the incidence and risk factors for evolution of AA into AML, and to administer salvage therapy for transformation, in time, during follow-up. In addition, the best supportive care must be fully integrated with diagnosis and treatment. For example, multiple anti-human leukocyte antigen (HLA) antibodies must be detected prior to the initiation of chemotherapy and appropriate, unrelated HLA-matched platelet donors must be selected prior to therapy in patients who were refractory to platelet transfusions (17). In conclusion, an increased number of factors must be considered when determining the most appropriate management of such secondary leukemia. This remains an unsatisfactory area with the greatest clinical challenge in secondary AML.

## References

[1] Ohara A, Kojima S, Hamajima N (1997). Myelodysplastic syndrome and acute myelogenous leukemia as a late clonal complication in children with acquired aplastic anemia. Blood.

[2] Barrett J, Saunthararajah Y, Molldrem J (2000). Myelodysplastic syndrome and aplastic anemia: distinct entities or diseases linked by a common pathophysiology?. Semin Hematol.

[3] Tichelli A, Gratwohl A, Nissen C, Speck B (1994). Late clonal complications in severe aplastic anemia. Leuk Lymphoma.

[4] Barrios NJ, Kirkpatrick DV, Levin ML, Varela M (1991). Transient expression of trisomy 21 and monosomy 7 following cyclosporin A in a patient with aplastic anemia. Leuk Res.

[5] Locasciulli A, Arcese W, Locatelli F, Di Bona E, Bacigalupo A, Italian Aplastic Anaemia Study Group (2001). Treatment of aplastic anaemia with granulocyte-colony stimulating factor and risk of malignancy: Italian Aplastic Anaemia Study Group. Lancet.

[6] Imashuku S, Hibi S, Bessho F (2003). Detection of myelodysplastic syndrome/acute myeloid leukemia evolving from aplastic anemia in children, treated with recombinant human G-CSF. Haematologica.

[7] Gurion R, Gafter-Gvili A, Paul M (2009). Hematopoietic growth factors in aplastic anemia patients treated with immunosuppressive therapy - systematic review and meta-analysis. Haematologica.

[8] Kojima S, Ohara A, Tsuchida M (2002). Risk factors for evolution of acquired aplastic anemia into myelodysplastic syndrome and acute myeloid leukemia after immunosuppressive therapy in children. Blood.

[9] Sloand EM, Yong AS, Ramkissoon S (2006). Granulocyte colony-stimulating factor preferentially stimulates proliferation of monosomy 7 cells bearing the isoform IV receptor. Proc Natl Acad Sci USA.

[10] Socie G, Mary JY, Schrezenmeier H, Marsh J (2007). Granulocyte-stimulating factor and severe aplastic anemia: a survey by the European Group for Blood and Marrow Transplantation (EBMT). Blood.

[11] Wang HF, Cheng YZ, Wang HP, Chen ZM, Lou JY, Jin J (2009). CD19-positive acute myeloblastic leukemia with trisomy 21 as a sole acquired karyotypic abnormality. J Zhejiang Univ Sci B.

[12] Langebrake C, Creutzig U, Reinhardt D (2005). Immunophenotype of Down syndrome acute myeloid leukemia and transient myeloproliferative disease differs significantly from other diseases with morphologically identical or similar blasts. Klin Padiatr.

[13] Udayakumar AM, Pathare AV, Muralitharan S, Alghzaly AA, Alkindi S, Raeburn JA (2007). Trisomy 21 as a sole acquired abnormality in an adult Omani patient with CD7- and CD9-positive acute myeloid leukemia. Arch Med Res.

[14] Cortes JE, Kantarjian H, O’Brien S (1995). Clinical and prognostic significance of trisomy 21 in adult patients with acute myelogenous leukemia and myelodysplastic syndromes. Leukemia.

[15] Wan TS, Au WY, Chan JC, Chan LC, Ma SK (1999). Trisomy 21 as the sole acquired karyotypic abnormality in acute myeloid leukemia and myelodysplastic syndrome. Leuk Res.

[16] Wang H, Ni W, Chen Z (2008). Clinical and cytogenetic features of hematologic malignancies associated with acquired trisomy 21. Zhonghua Yi Xue Yi Chuan Xue Za Zhi.

[17] Xia WJ, Ye X, Tian LW (2010). Establishment of platelet donor registry improves the treatment of platelet transfusion refractoriness in Guangzhou region of China. Transfus Med.

